# Clinical Impact of High Throughput Sequencing on Liquid Biopsy in Advanced Solid Cancer

**DOI:** 10.3390/curroncol29030155

**Published:** 2022-03-10

**Authors:** Etienne Gouton, Nausicaa Malissen, Nicolas André, Arnaud Jeanson, Annick Pelletier, Albane Testot-Ferry, Caroline Gaudy-Marqueste, Laetitia Dahan, Emeline Tabouret, Thomas Chevalier, Laurent Greillier, Pascale Tomasini

**Affiliations:** 1 Department of Early Phase Cancer Trials Center «CLIP2», CHU Timone, Assistance Publique Hôpitaux de Marseille, Aix-Marseille University, 13005 Marseille, France; etienne.gouton@gmail.com (E.G.); nausicaa.malissen@ap-hm.fr (N.M.); nicolas.andre@ap-hm.fr (N.A.); arnaud.jeanson@ap-hm.fr (A.J.); annick.pelletier@ap-hm.fr (A.P.); albane.testot-ferry@ap-hm.fr (A.T.-F.); caroline.gaudy@ap-hm.fr (C.G.-M.); laetitia.dahan@ap-hm.fr (L.D.); emeline.tabouret@ap-hm.fr (E.T.); thomas.chevalier@ap-hm.fr (T.C.); laurent.greillier@ap-hm.fr (L.G.); 2 Dermatology and Skin Cancer Department, Aix Marseille University, APHM, CRCM Inserm U1068, CNRS U7258, CHU Timone, 13005 Marseille, France; 3 SMARTc Unit, Centre de Recherche en Cancérologie de Marseille, Inserm U1068, Aix Marseille University, 13005 Marseille, France; 4 Department of Digestive Oncology, CHU Timone, Assistance Publique Hopitaux de Marseille, Aix-Marseille University, 13005 Marseille, France; 5 Institute de Neurophysiopathol, Aix-Marseille University, APHM, CNRS, INP, Service de Neuro-Oncologie CHU Timone, 13005 Marseille, France; 6 Department of Medical Oncology, CHU Timone, Assistance Publique Hopitaux de Marseille, Aix-Marseille University, 13005 Marseille, France; 7 Multidisciplinary Oncology & Therapeutic Innovations Department, Aix Marseille University, CNRS, INSERM, CRCM, APHM, 13015 Marseille, France

**Keywords:** cancer, molecular profiling, liquid biopsy, targeted therapy, precision oncology

## Abstract

Background: Cancer therapies targeting actionable molecular alterations (AMA) have developed, but the clinical routine impact of high-throughput molecular profiling remains unclear. We present a monocentric experience of molecular profiling based on liquid biopsy in patients with cancer. Methods: Patients included had solid cancer and underwent cfDNA genomic profiling with FoudationOne Liquid CDx (F1LCDx) test, analyzing 324 genes. Primary endpoint was to describe patients with an AMA for whom clinical decisions were impacted by F1LCDx test results. Results: 191 patients were included, mostly with lung cancer (46%). An AMA was found in 52%. The most common molecular alterations were: TP53 (52%), KRAS (14%) and DNMT3 (11%). The most common AMA were: CHEK2 (10%), PIK3CA (9%), ATM (7%). There was no difference in progression-free survival (2.66 months vs. 3.81 months, *p* = 0.17), overall survival (5.3 months vs. 7.1 months, *p* = 0.64), or PFS2/PFS1 ratio ≥ 1.3 (20% vs. 24%, *p* = 0.72) between patients receiving a molecularly matched therapy (MMT) or a non-MMT, respectively. Patients with a MMT had an overall response rate of 19% and a disease control of 32%. Conclusions: Routine cfDNA molecular profiling is feasible and can lead to the access of targeted therapies. However, no notable benefit in patient’s outcomes was shown in this unselected pan-cancer study.

## 1. Introduction

Oncogenesis is a complex process due to the accumulation of molecular alterations (MA) involved in the regulation of hallmarks of cancers [1]. Historically, the standard testing methodology for oncogenic drivers has been single-gene testing methods. However, recently, the development of high-throughput molecular profiling, particularly next-generation sequencing (NGS), has led to a rapid improvement in diagnostic testing performance. Current techniques provide an overview of the genomic complexity of primary tumors within a timeframe in accordance with routine practice and at an affordable cost. Such advances in sequencing technologies have permitted the identification of genomic alterations that can act as predictive biomarkers or therapeutic targets, which therefore, can have major clinical impact [2].

Approximately 400 to 500 oncogenic drivers have been described across various cancer types [3,4], although not all are clinically relevant. Only a small number can be targeted by available specific therapies. Recent breakthroughs in genomic findings have led to the development of molecularly matched therapies (MMT), which are directed against the abnormal proteins resulting from such molecular alterations. In contrast, cytotoxic chemotherapy inhibits cell replication or induces DNA damage. Several studies have shown that targeting various molecular alterations of interest in specific tumor types can improve outcomes, including *EGFR*, *ALK*, *ROS1* or *BRAF V600E* in non-small-cell lung cancer [5,6,7,8], *BRAF V600E* in melanoma [9], *BCR-ABL* in chronic myeloid leukemia [10], or *RET* in medullary thyroid cancer [11].

Whereas some oncogenic drivers occur with a high frequency in specific cancers, some other targetable drivers are found at a lower frequency across multiple tumor types and pathologies [12]. This observation was the starting point for challenging the historical strategy of histology-based treatments. Some MMT-associated outcomes (i.e., response rates; survival rates) were evaluated regardless of the tumor type with high responses rates and good survival outcomes, such as Entrectinib in *NTRK* fusion-positive solid tumors, Vemurafenib in non-melanoma *BRAF V600* cancers, or even immune checkpoint-inhibitors as Pembrolizumab in MSI-high tumors [13,14,15]. This recent approach is the basis of precision medicine in oncology, according to which treatments are gene-directed and individualized for each patient regardless of the pathology [16].

So far, the real benefit of precision oncology remains unclear. Many retrospective and non-randomized prospective studies suggested a clinical improvement of individualized MMT over standards of care [17,18,19,20,21,22,23,24]. However, the only randomized phase 2 study failed to show a benefit of the MMT strategy, with a median progression-free survival (PFS) of 2.3 months in the experimental group versus 2.0 months in the control group (*p* = 0.41) [25]. Hypotheses to explain these results could be spatial and temporal intratumor heterogeneity, or molecular differences between primary tumor and metastases that occur under multiple selective pressures (treatments, immune system) [26,27]. Considering this heterogeneity, it appears fundamental to determine a “real-time” comprehensive genomic profile before therapy initiation, ensuring the best therapy choice. Iterative NGS is a valid option but may require repeated tissue biopsies that can be difficult for patients.

Circulating cell-free DNA (cfDNA)-analysis, also referred to as a liquid biopsy, is a biomarker analysis tool that uses multiple body fluids (blood, urine, cerebrospinal fluid) to diagnose and monitor cancer without limitations and the inconvenience of tissue biopsies [28]. Using cfDNA has many advantages, such as reduced time from sample to results compared to a tumor tissue analysis [29], and providing a better reflection of the systemic tumor burden and intratumoral heterogeneity that can be missed by single-site tissue biopsies [30,31]. Moreover, patient’s acceptability of iterative blood samples may be better, with less complications.

In the present study, we aimed to evaluate the feasibility and clinical utility of high-throughput molecular profiling based on a liquid biopsy in patients with advanced solid cancers, by assessing the proportion of patients treated with an MMT and their outcomes.

## 2. Materials and Methods

### 2.1. Study Design, Endpoints, and Patients

This was a retrospective, monocentric study conducted at the Assistance Publique–Hôpitaux de Marseille (AP-HM, Marseille, France). The primary endpoint was to describe the proportion of patients with an actionable molecular alteration (AMA) for whom clinical decisions were impacted by the results of circulating cfDNA comprehensive profiling. The secondary endpoints were to assess the proportion of patients with solid cancer who presented molecular alterations and AMA, PFS2 (defined as the progression-free survival on the first therapy after molecular profiling (treatment 2), response rates on treatment 2, overall survival (OS), PFS2/PFS1 ratio (PFS1 being the progression-free survival for the last therapy prior to molecular profiling (treatment 1)). A PFS2/PFS1 ratio ≥ 1.3 was considered as a benefit of treatment 2 for the patient, as defined by Von Hoff et al. [32]. 

Patients were eligible if they had a solid tumor and underwent comprehensive cfDNA genomic profiling during routine care in our centre (AP-HM), between February 2019 and March 2021. No restriction on tumor type, pathology, previous treatments, or other factor were imposed. cfDNA genomic profiling was performed in patients selected at the physician’s discretion. For each patient, the following clinical data at the time of the molecular profiling were retrospectively collected in electronic medical records: demographic data (age, sex, smoking status), biological data (tumor type, molecular status), radiological data (tumor stage, response to treatments according to RECIST 1.1), treatment data (previous and subsequent treatment regimen, time on treatment(s)) and outcome data (progression-free survival, overall survival). Every patient provided a signed consent form permitting the use of these data for research purpose. This non-interventional retrospective study was performed under the approval of our institution (registration number: PADS20-341).

### 2.2. Genomic Analyses and Targeted Therapies

Genomic analyses were performed using a FoundationOne Liquid CDx (F1LCDx) test, an NGS-based in vitro diagnostic tool analyzing a panel of 324 genes, using circulating cell-free DNA isolated from plasma derived from anti-coagulated peripheral whole blood. Blood samples were collected in two tubes of whole blood (8.5 mL per tube), using FoundationOne Liquid CDx Specimen Collection and Shipping Kit. Samples were then shipped at an ambient temperature to Foundation Medicine, Inc. (Cambridge, MA, USA). The F1LCDx assay employs a single DNA extraction method to obtain cfDNA from plasma of whole blood. Genomic signature such as a tumor mutational burden (TMB), microsatellite instability (MSI) and tumor fraction were also reported. Technical information of the F1LCDx test are available in the FDA label [33]. 

Actionable molecular alterations (AMA) were defined as molecular alteration for whom a therapy targeting either the molecular alteration or the pathway activated by the molecular alteration was available, using the OncoKB database (v3.4; 17 June 2021) [34]. All evidence levels from 1 to 4 were considered (level 1: FDA-recognized biomarker predictive of response to an FDA-approved drug in a specific cancer type; level 2A: standard care biomarkers predictive of response to an FDA-approved drug in a specific cancer type; level 2B: standard care biomarkers predictive of response to an FDA-approved drug in another cancer type; level 3A: compelling clinical evidence in a specific cancer type supporting the biomarker as predictive of response to a drug that is not yet approved in the standard of care; level 3B: compelling clinical evidence in another cancer type supporting the biomarker as predictive of response to a drug that is not approved in the standard of care; and level 4: compelling biological evidence supporting the non-FDA-recognized biomarker as predictive of response to a drug). Biomarkers predictive of a response in haematological pathologies or predictive of therapy-resistance were not considered. TMB was considered “TMB-high” when the score was ≥10 mutations/Mb. No distinction between somatic vs. germline alterations were provided by the test. However, our molecular tumor board reviewed the test results that were submitted to it, and compared them with somatic genomic databases.

### 2.3. Statistical Analysis

Descriptive statistics were used to analyze patient’s characteristics and molecular profiling. Responses while on treatment were evaluated according to RECIST 1.1 and the RANO criteria for neuro-oncological tumors [35,36]. The objective response rate (ORR) was defined as the percentage of complete responses (CR) and partial responses (RP). The disease control rate (DCR) was defined as the percentage of complete responses, partial responses, and stable diseases (SD). OS was defined as the time from the start of treatment 2 to death from any cause, censored at the date of the last follow-up. Patients who did not received treatment 2 were excluded from OS analyses. Furthermore, PFS2 was defined as the time from the start of treatment 2 to progression (according to RECIST 1.1 and RANO criteria for neuro-oncological tumors) or death from any cause, censored at the date of last follow-up, while PFS1 was defined as the time from the start of treatment 1 to progression (according to RECIST 1.1) or death from any cause, censored at the date of last follow-up. 

A comparison of continuous variables was performed using the non-parametric Mann–Whitney U test, whereas Fisher’s exact test was used to compare categorical variables. Survival analyses were estimated with the Kaplan–Meier method and group survivals were compared using the log-rank test. A univariate Cox regression model was applied to determine the hazard ratio (HR). Concerning response rates, a binary logistic regression was used to calculate the Odds Ratios (OR). All statistical tests were two-sided, with a confidence interval of 95%, and a *p*-value < 0.05 was considered significant. Statistical analyses were performed on IBM SPSS Statistics software, version 28.0.0.0 (IBM SPSS Inc., Chicago, IL, United States of America.

## 3. Results

### 3.1. Patients

**Figure 1:**
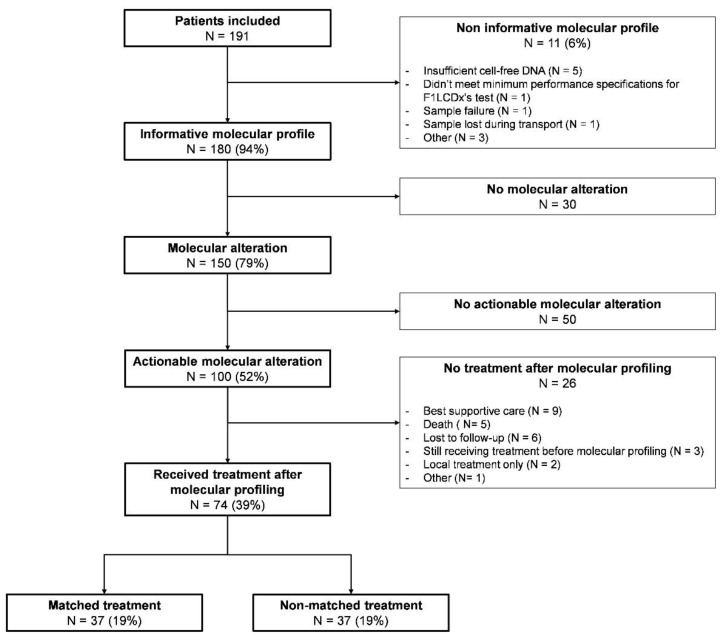
Study flow chart. F1LCDx: FoundationOne Liquid CDx.

The study flow chart is reported in Figure 1. Between February 2019 and March 2021, 191 patients underwent a F1LCDx test, among which 11 patients (6%) had a non-informative test, mostly because of insufficient cell-free DNA (*n* = 5), thus leaving 180 patients (94%) with an exploitable molecular profiling. An AMA was found in 100 patients (52%), but only 74 patients (39%) received a subsequent treatment after molecular testing (treatment 2). Among them, 37 patients (19%) were treated in each group, either with a MMT or a non-MMT.

Patient’s characteristics are described in Table 1. Patients had a good general status, with an ECOG PS of 0 or 1 (84%). The most common tumor types were lung (46%), melanoma (11%), breast (10%) and pancreatic cancers (6%) (Figure S1, Supplementary Materials). Pathological types were mostly adenocarcinomas (64%), melanoma (11%), squamous cell carcinomas (7%) and other carcinomas (8%). PD-L1 status was available for only 76 patients (40%), mostly lung cancer, with 21%, 10%, and 8% of patients having a PD-L1 expression of 0%, 1 to 49%, and 50 to 100%, respectively. They were heavily pre-treated: 26% of patients received at least four prior lines of treatments, with a median of three prior lines of systemic therapy (range: 0–10). Previous treatments were cytotoxic chemotherapy (81%), immune checkpoint-inhibitors (50%), targeted therapy (37%), antiangiogenic treatments (21%) and hormonotherapy (10%). Patients received a median of 1 subsequent therapy after F1LCDx test (range: 0–4), which consisted in chemotherapy (49%), targeted therapies (35%), immune checkpoint-inhibitors (14%), antiangiogenic (8%) or hormonotherapy (6%). Four patients (3%) had radiotherapy and one patient (1%) underwent surgery.

### 3.2. Molecular Profiling and Treatments

The characteristics of molecular profiling with F1LCDx assay are reported in Table 2. Among the 180 patients in which an exploitable molecular profile was obtained, results of the F1LCDx test were received in a median of 13 days (range: 5–38). Most of the genomic analyses were performed after progression with a non-targeted therapy (61%), during treatment (24%), after progression with a targeted therapy (6%), or at diagnosis (4%). Patients had a median of two molecular alterations (range: 0–18). The median number of AMA per patient was 1 (range: 0–5). The median percentage of tumor fraction was 21% (range: 10–72), and MSI status was “MSI-high” in only one patient with metastatic lung adenocarcinoma. TMB was available in 96 patients (53%) and was considered “TMB-high” in 6%. When comparing patients with or without AMA, there were significantly more “TMB-high” patients in the AMA group (11% vs. 1%; *p* = 0.01). Overall, 82% of patients already had a NGS prior to our F1LCDx test, but remarkably, 54% of patients in the AMA group did not have any previously known AMA. Fifteen (23%) patients with an AMA in previous NGS had no AMA with F1LCDx test, thus were sorted into the “non-AMA” group in our analysis. Previous NGS were performed on a solid tumor biopsy in 142 patients (97%) (primary tumor in 86 patients (59%) and metastases in 56 patients (38%)), and on a liquid biopsy in four patients (3%). 

**Figure 2:**
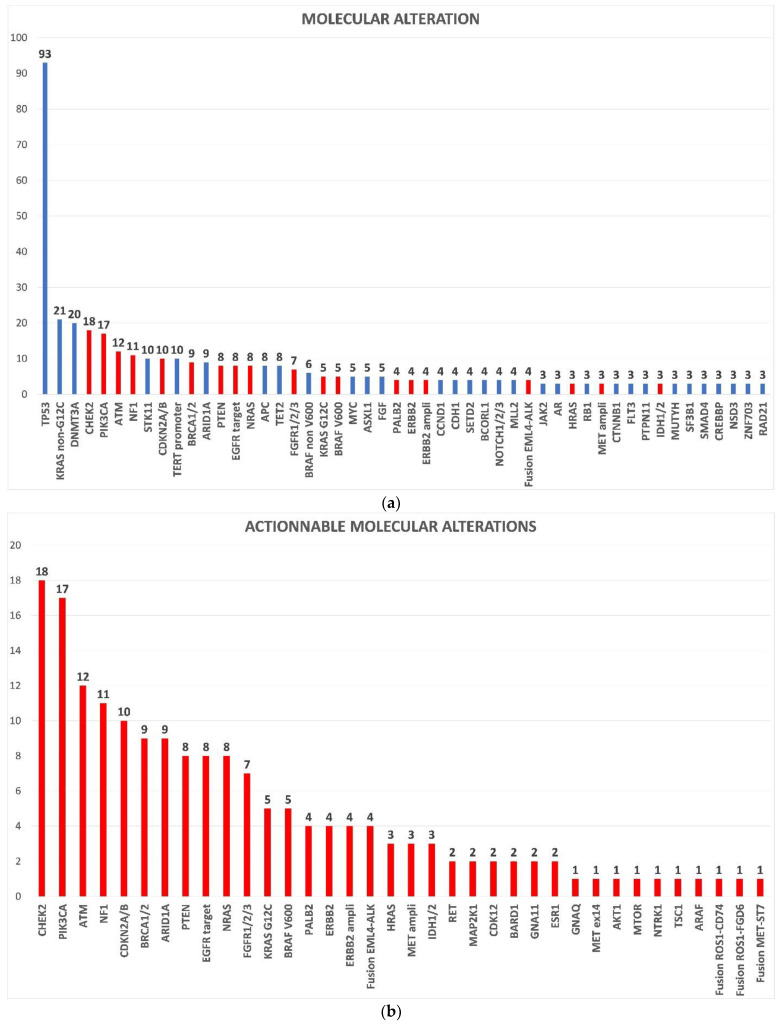
(**a**) 50 most frequent molecular alterations in the overall population and (**b**) actionable molecular alterations in the overall population. Actionable molecular alterations are colored in red. Non-actionable molecular alterations are colored in blue. Number of patients are provided above bars.

The 50 most frequent molecular alterations in all patients with an informative F1LCDx test (*n* = 180) are shown in Figure 2a; alterations of TP53 (52%), KRAS (14%) and DNMT3A (11%) being the most frequent. The most common AMA were (Figure 2b): CHEK2 (10%), PIK3CA (9%), ATM (7%), NF1 (6%), CDKN2A (6%), BRCA1/2 (5%), and ARID1A (5%) alterations. Figure S2 (Supplementary Materials) shows the proportion of molecular alterations according to the four most common tumor types (lung, breast, melanoma, and pancreatic cancers).

Among the 37 patients with an actionable molecular alteration and treated with an MMT, 33 patients (89%) received a targeted therapy (TT), three patients (8%) received an immune checkpoint-inhibitor (ICI) (including two patients with “TMB-high”, and one patient was treated with a combination of TT and ICI in a clinical trial for a CHEK2 alteration; none of them were previously treated with ICI), three patients (8%) received an hormonotherapy and only one patient (2%) was treated with an antibody-drug conjugate (trastuzumab-emtansine for an ERRB2 amplification). Those patients were treated according to the decision of our local molecular tumor board (MTB) for 16 patients (43%), according to drugs approval for 10 patients (27%), in a clinical trial for five patients (14%), and according to physician’s choice for two patients (5%). The most frequent targeted molecular alterations were as follows: *EGFR* mutation (14%), *ATM* mutation (8%), *BRAF* mutation (8%), *MET* alteration (8%; two amplifications and one exon 14 mutation), *PTEN* mutation (8%) and *PIK3CA* mutation (8%) (Table S1, Supplementary Materials). The median variant allelic frequency (VAF) of these targeted AMA was 6.8% (range: 0.15–50.9) and 11/37 patients (30%) had a VAF > 10%.

**Figure 3:**
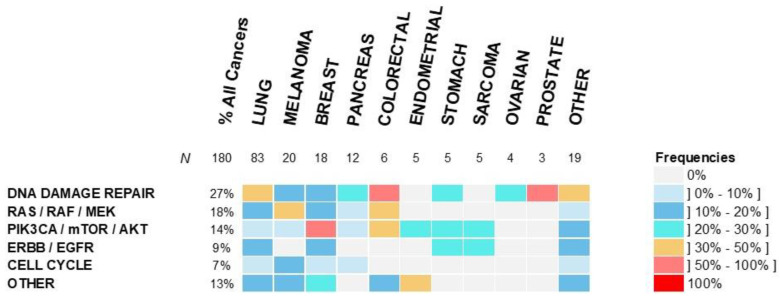
Incidence of molecular alterations according to molecular pathways and tumor type. Percentage of patients with an alteration of a molecular pathway per cancer type is color-coded as indicated on the labels on the right of the graph.

Considering molecular pathways, the DNA damage-repair system was the most frequently altered pathway in 27% of cases, followed by RAS/RAF/MEK and PIK3CA/mTOR/AKT pathway alterations, in 18% and 14% of patients, respectively (Figure 3 and Figure S3, Supplementary Materials). Patients with PIK3CA/mTOR/AKT pathway alterations were more frequently treated with an MMT than with a non-MMT (35% vs. 14%, respectively), but this difference was not significant (*p* = 0.06) (Table 1).

### 3.3. Efficacy

Among the 180 patients with an exploitable F1LCDx test, median OS was 9.12 months (95%CI: 8.02–10.21). A total of 127 patients were evaluable for PFS2, with a median of 2.95 months (95%CI: 2.23–3.67).

**Figure 4:**
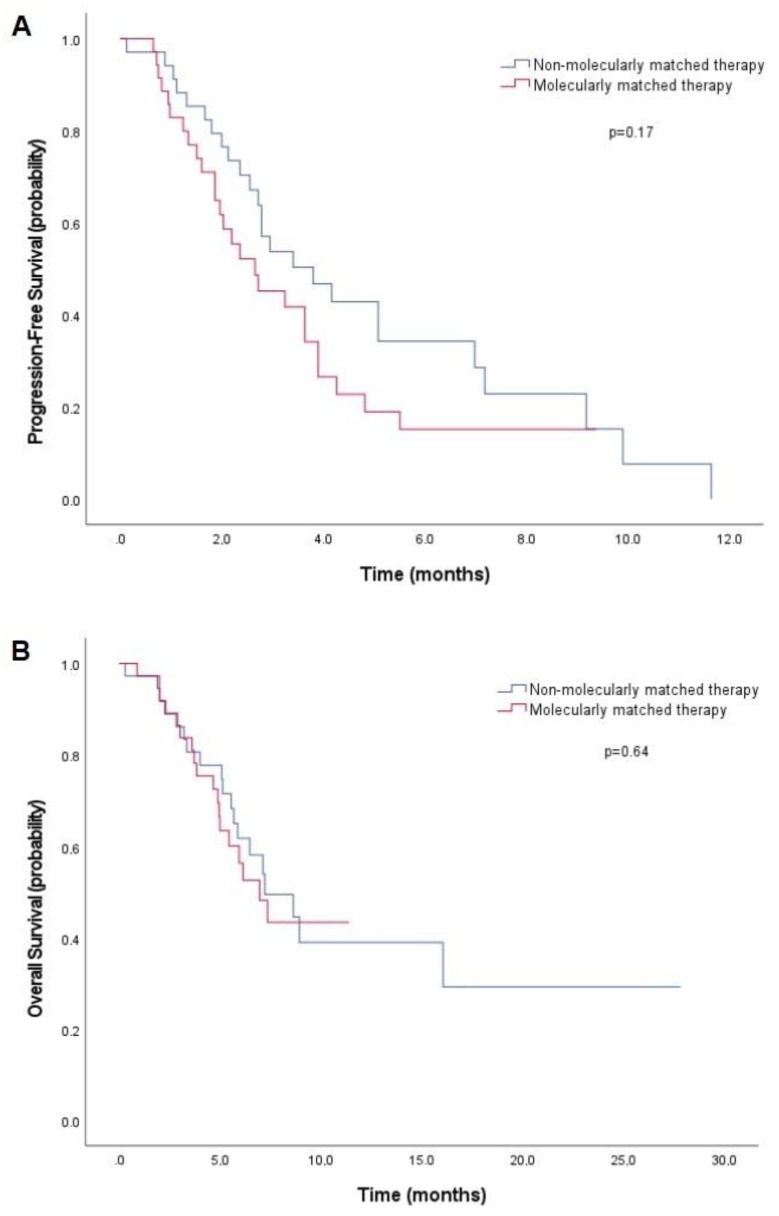
Kaplan–Meier curves of progression-free survival (PFS2) (**A**) and overall survival (**B**) in patients with actionable molecular alterations treated with “molecularly matched therapy” or “non-molecularly matched therapy”.

Among the 74 patients with an AMA who received a treatment after molecular profiling (treatment 2) (Table 3), there was no statistical difference in median PFS2 in the “MMT” and “non-MMT” groups (2.66 months [95%CI: 1.35–3.97] vs. 3.81 months [95%CI: 2.10–5.52], *p* = 0.17) (Figure 4A). OS was not different either, with a median OS of 5.3 months (95%CI: 2.8–7.8) in the “MMT” group, and 7.1 months (95%CI: 2.2–11.9) in the “non-MMT” group (*p* = 0.64) (Figure 4B).

Response to treatment 2 was evaluable in 54 patients (29 patients in the “MMT” group, and 26 patients in the “non-MMT” group) (Table 3). One patient with lung cancer and EML4-ALK fusion treated with alectinib had a complete response. The overall response rate (ORR) was 16% and disease control was 41%. When comparing the two subgroups, no statistical difference in ORR (19% vs. 16%; OR = 1.11 [95%CI: 0.32–3.88], *p* = 0.87) and DCR (32% vs. 49%; OR = 0.33 [95%CI: 0.11–1.02], *p* = 0.05) was seen among patients with “MMT” and “non-MMT” patients, respectively. 

**Figure 5:**
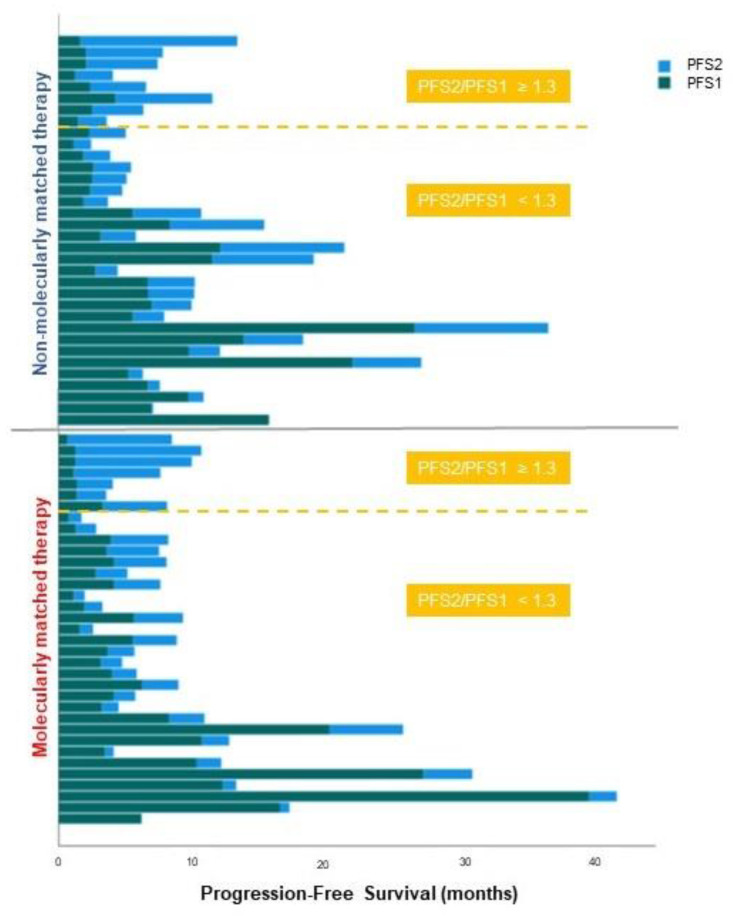
Duration of PFS1 and PFS2 according to the treatment regimen, ordered by decreasing PFS2/PFS1 ratio. Patients above the yellow line have a PFS2/PFS1 ratio ≥ 1.3.

The median PFS2/PFS1 ratio was 0.63 (range: 0–11.9) in the “MMT” group vs. 0.84 (range: 0–7.2) in the “non-MMT” group, with a ratio ≥ 1.3 in 7/35 patients (20%) and 8/34 patients (24%), respectively (OR = 0.81 [95%CI: 0.26–2.56], *p* = 0.72) (Table 3 and Figure 5). The PFS2/PFS1 ratio ≥ 1.3 was significantly associated with a better OS, with a hazard ratio (HR) for death of 0.32 (95%CI: 0.12–0.84; *p* = 0.02) (Figure S4, Supplementary Materials).

In an additional subgroup analysis, in the cohort (*n =* 50) of patients with a level 1–2 AMA (according to the OncoKB database) that received treatment after F1LCDx testing (treatment 2), no clinical benefit of the MMT strategy was found compared to non-MMT in terms of PFS2 (2.7 months vs. 2.8 months, *p* = 0.87), OS (4.7 months vs. 7.2 months, *p* = 0.6), the PFS2/PFS1 ratio ≥ 1.3 (19% vs. 27%, *p* = 0.73), or tumor responses (Table S2, Supplementary Materials).

## 4. Discussion

This study is a real-world experience of high-throughput molecular profiling based on liquid biopsy in patients with solid cancers. An AMA was found in 52% of the 191 patients in our centre who underwent a FoundationOne Liquid CDx test regardless of tumor type. Our study found no difference in terms of clinical outcomes in patients treated according to F1LCDx test results, with a molecularly matched treatment in comparison with non-molecularly matched treatments.

In the past years, many retrospective and prospective studies demonstrated mixed results with treatments matched to the molecular profiling results [16]. In the prospective MOSCATO trial, 948 patients were included and biopsied, and an AMA was found in 49% of patients [20]. Among the 199 patients treated with a MMT, the PFS2/PFS1 ratio was ≥ 1.3 in 33% of patients. An overall response rate of 11% and a disease control rate of 63% were found, and the molecularly matched strategy was considered as promising in patients with hard-to-treat and heavily pre-treated cancer. Similarly, in a recent publication, the PERMED-01 study showed an interesting overall response rate and disease control rate in the “matched therapy” group (19% and 35%, respectively), but there was no difference with the “non-matched therapy” group (25% and 34%, respectively) [37]. In the group treated with MMT, a PFS2/PFS1 ratio ≥ 1.3 was found in 36% of patients (versus 20% in the “non-matched” population). This percentage of PFS2/PFS1 ratio ≥ 1.3 was similar in several prospective non-randomized studies: 33% in MOSCATO trial [20], 25% in EXOMA study [38], 42% in PROFILE study [23], and 45.3% in PREDICT study [17].

In comparison with these previous trials, we report here a comparable ORR and DCR of 19% and 32%, respectively, in the “matched” group (vs. 16% and 49%, respectively, in the “non-matched’ group). The percentage of PFS2/PFS1 ratio ≥ 1.3 was lower in our cohort, with 20% in the “MMT” group and 24% in “non-MMT” group. This may be explained by patients being treated in a very late-stage disease, with a median of only 1 subsequent therapy after an F1LCDx test (including the MMT). Furthermore, among the 74 patients with AMA who received treatment after the F1LCDx test, 73% did not receive any further therapy, supporting the hypothesis that our patients were at an end-stage of their therapeutic strategy, and that molecular profiling may not bring any benefit to these fragile patients. We found that a PFS2/PFS1 ratio ≥ 1.3 was statistically associated with improved OS (HR = 0.32, *p* = 0.02), which was consistent with previous studies [32,37]. No difference was found in terms of PFS2, PFS2/PFS1 or OS between the “MMT” and “no-MMT” groups in our study. This was consistent with the EXOMA study [38], but different to PERMED-01 in which “matched” patients displayed a more frequent PFS2/PFS1 ratio ≥ 1.3 than “non-matched” patients (36% vs. 20%, *p* = 0.013) [37]. In the PREDICT study, patients treated with “matched therapy” also had a better PFS2 (4.0 months vs. 3.0 months, *p* = 0.039) and PFS2/PFS1 ratio ≥ 1.3 (45.3% vs. 19.3%, *p* = 0.004) [17].

The randomized SHIVA phase 2 trial was conducted between 2012 and 2014 [25]. A total of 195 patients with an AMA were randomly assigned to receive either a molecularly targeted agent or a treatment recommended by the physician. No difference was found in terms of PFS between the experimental and control groups (2.3 months vs. 2.0 months, respectively), similarly to our study. In addition, there was no difference either in ORR (4.1% vs. 3.4%, in experimental and control groups, respectively). Authors notably explained these negative results by the small number of targeted therapies available in this study, which did not cover all molecular alterations found at the time. Indeed, only patients with molecular alterations that matched one of the 11 available MMT were included, thus excluding patients with other AMA which could have been targeted by another off-label therapy. However, despite over-selected AMA and targeted agents that should have favored the “matched” patients, these negative results are consistent with ours.

In our cohort, only 37% of the patients included have been treated according to the result of molecular profiling. This number is similar to previous studies reporting a range from 5% to 49%, with a median of 23% [17,18,19,20,21,22,23,24,37,39]. There are several hypotheses to explain the small number of patients actually treated with MMT: (i) the delays for F1LCDx test results may be considered as too long by the physicians, (ii) molecular profiling may have been performed in a very advanced stage of disease in patients with poor performance status, (iii) the treating physician may lack of experience in using NGS, (iv) poor access to molecular tumor board, (v) poor access to off-label targeted therapies, early access programs and clinical trials.

Additionally, homologous recombination deficiency (HRD) is driven by the inactivation of numerous genes involved in DNA double-strand damage repair, including BRCA1/2, PALB2 or CHEK2. A loss of function of these repairing proteins can lead to increased molecular alterations and therefore, to cancer. The detection of HRD is clinically significant, as recent therapies such as PARP inhibitors (PARPi) can be proposed [40]. HRD-score is an effective tool in evaluating genomic instability, using three DNA-based measures (loss of heterozygosity, telomeric allelic imbalance, and large-scale state transitions) [41]. Several studies similar to ours have shown that HRD is a very common altered pathway in cancer. A high HRD-score was found in 41% of patients in the PERMED-01 study [37] and in 35% in another pan-cancer analysis [41], in which breast and ovarian cancer were the most frequent tumor types. In the present study, although the F1LCDx test did not provide us with an HRD-score, an alteration of genes involved in the DNA damage repair system was seen in 27% of patients. Although it was the most frequently altered pathway, the predominance of lung cancer can explain this smaller proportion in our study: lung cancer displays a lower frequency of HRD gene alteration compared to breast or ovarian tumors [42]. Surprisingly, a great number of CHEK2 alterations were found (10% of exploitable samples), being the most frequent actionable molecular alteration found mostly in lung cancer. This proportion was greater in our study than previously reported in the literature, as it is usually found in 0.6% to 4.3%, depending on tumor type [43]. Two patients (one lung cancer and one stomach cancer) were treated with a CHEK2-targeted PARP-inhibitor: one patient had a stable disease followed by rapid progression, and one patient experienced progressive disease at first tumor evaluation. This was consistent with previous reports on the inefficiency of PARPi in CHEK2-altered prostate cancer [44,45].

To our knowledge, this study is the first to evaluate the feasibility and clinical impact of liquid biopsy for cfDNA-based NGS analysis in pan-cancer patients. Previous studies similar to ours have been conducted with tissue samples, while other studies using liquid biopsy were limited to specific cancer types, mostly lung cancer. Nowadays, the analysis of cfDNA extracted from plasma is well established in non-small cell lung cancer international practice guidelines [46,47], mostly for disease monitoring, with a specificity of almost 100% [48]. This strategy provides a number of advantages compared to standard tissue biopsy [49,50], such as being a non-invasive technique with better tolerability for the patient, reproducibility. High cfDNA levels seems to correlate with negative impact on PFS and OS. Moreover, cfDNA provides a better reflection of tumor molecular heterogeneity and facilitates the detection of acquired treatment resistance mechanisms. However, in our cohort, the 37 patients with a targeted AMA had relatively low variant allelic frequencies (VAF), with 14% and 30% having a VAF < 1% or >10%, respectively. The hypothesis is that a low VAF could represent very subclonal AMA, and therefore has little therapeutic impact, but could also represent a lower sensitivity of the cfDNA test [48,51]. Nevertheless, despite this lower sensitivity of the liquid biopsy, we found similar results to the tissue analysis in the present study. This supports the use of cfDNA, because of its greater simplicity with comparable clinical outcomes. Finally, in our study, discrepancies between F1LCDx results and previous molecular profiles can be explained by differences in the gene panels, with F1LCDx having a larger number of genes analyzed. This supports the 54% rate of newly discovered AMA, which did not appear during previous NGS testing.

This study has some limitations. First, this was a retrospective and monocentric cohort, carrying all the bias and limitations associated with this type of analysis. Nonetheless, the real-world design of our study can offer data with a real clinical impact, as no restriction on any characteristics was mandatory for inclusion. Second, as patients who underwent the F1LCDx cfDNA testing were only selected at the physician’s choice, heavily pre-treated patients who had already received standard therapies could have preferentially been elected, thus leading to a selection bias. Third, the lack of differences in outcomes between patients treated with “matched” or “non-matched” therapy can be explained by the small number of subjects in each group. Our patients were heavily pre-treated, supporting the notion that precision medicine oncology should be preferred in an early stage disease. Finally, the definition of the actionability of a molecular alteration was based on the OncoKB database, as recommended in European guidelines [52]. However, there was no systematic molecular tumor board assessment to facilitate the interpretation of molecular testing results, and to help the decision of the most appropriate therapeutic strategy. Several studies have shown the impact and importance of MTB in treatment decisions, patient’s outcomes and clinical trial enrolment [53,54,55].

The results of this study may transform clinical practices in several ways. We showed that high-throughput molecular profiling is feasible in a clinical routine with liquid biopsy, with good performance. Moreover, consistent outcomes were found in our liquid biopsy-based analysis compared to previous studies in the literature based on tissue molecular analyses, enlightening the possibility of using less-invasive sampling. Such an argument is supported by the fact that most of the patients do not seem to benefit from molecular profiling. Therefore, non-invasive techniques should be the best option in this situation. However, this molecularly matched strategy can spectacularly change outcomes only in a small proportion of patients. To maximise the efficiency of molecular profiling, it appears important to realize it in an early stage disease strategy. Systematic molecular tumor board discussions should also be systematically proposed.

## 5. Conclusions

In summary, our study did not reveal the clinical benefit of a molecularly matched treatment strategy in a pan-cancer cohort. However, some patients may benefit from such a personalized therapy based on histologic, molecular, or clinical attributes. An analysis of these responder patients may help to understand the biological factors of the treatment efficiency. In the era of precision oncology, it appears fundamental to find new ways or technologies to better select the right treatment for the right patient, and at the right time.

## Figures and Tables

**Table 1:** Patient’s characteristics.

Patient’s Characteristics	Overall Population *n* = 191	AMA + MMT *n* = 37 (19%)	AMA + Non-MMT *n* = 37 (19%)	*p*-Value *
Age				0.1
Median, years (range)	61 (15–88)	57 (24–88)	61 (45–78)	
Sex				0.35
Female	107 (56%)	19 (51%)	13 (35%)	
Male	84 (44%)	18 (49%)	24 (65%)	
ECOG PS				0.63
0	67 (35%)	10 (27%)	11 (30%)	
1	93 (49%)	21 (57%)	23 (62%)	
≥2	31 (16%)	6 (16%)	3 (8%)	
Smoking Status				0.81
Smoker (active or former)	94 (49%)	18 (49%)	19 (51%)	
Missing	17 (9%)	2 (5%)	3 (8%)	
Tumor Type				0.84
Lung	88 (46%)	18 (49%)	17 (46%)	
Melanoma	21 (11%)	6 (16%)	5 (14%)	
Breast	19 (10%)	7 (19%)	4 (10%)	
Pancreas	12 (6%)	1 (3%)	3 (8%)	
Sarcoma	7 (4%)	0	1 (3%)	
Colorectal	7 (4%)	1 (3%)	2 (5%)	
Other	37 (19%)	4 (10%)	5 (14%)	
Extension Stage				1
Metastatic	180 (94%)	36 (97%)	35 (94%)	
Other	11 (6%)	1 (3%)	2 (5%)	
Previous Systemic Treatment				0.15
Median (range)	3 (0–10)	3 (0–9)	3 (0–7)	
Missing	1	0	0	
Subsequent Systemic Treatment				0.73
Median (range)	1 (0–4)	1 (1–3)	1 (1–3)	
Missing	32	0	0	
Molecular Pathways Altered				
DNA damage repair system	-	15 (41%)	18 (49%)	0.64
RAS/RAF/MEK	-	14 (38%)	10 (27%)	0.46
PIK3CA/mTOR/AKT	-	13 (35%)	5 (14%)	0.06
ERBB/EGFR	-	10 (27%)	5 (14%)	0.25
Cell cycle	-	3 (8%)	5 (14%)	0.71
Other	-	11 (30%)	5 (14%)	0.16

MA: actionable molecular alteration; MMT: molecularly matched therapy. * *p*-value is provided for comparison between groups: “AMA + MMT” vs. “AMA + non-MMT”.

**Table 2:** Molecular profiling’s characteristics.

F1LCDx’s Characteristics	Overall Population *n* = 180	AMA *n* = 100 (52%)	Non-AMA *n* = 80 (42%)	*p*-Value *
Result Delay				0.56
Median, days (range)	13 (5–38)	13 (5–28)	13 (7–27)	
Timing of F1LCDx testing				0.0004
Progression on non-TT	109 (61%)	74 (74%)	35 (44%)	
During treatment	44 (24%)	16 (16%)	28 (35%)	
Progression on TT	11 (6%)	6 (6%)	5 (6%)	
Diagnosis	7 (4%)	1 (1%)	6 (7%)	
Metastatic relapse	6 (3%)	2 (2%)	4 (5%)	
Other	3 (2%)	1 (1%)	2 (3%)	
Previous Molecular Profile				
n (%)	147 (82%)	83 (83%)	64 (80%)	-
with previous MA	99/147 (67%)	60/83 (72%)	39/64 (61%)	0.7
with previous AMA	53/147 (36%)	38/83 (46%)	15/64 (23%)	0.16
Tumor Fraction (%)				0.91
Median (range)	21 (10–72)	21 (10–72)	19 (11–71)	
Tumor Mutational Burden				0.01
<10 mut/Mb	84 (47%)	43 (43%)	41 (51%)	
≥10 mut/Mb	12 (6%)	11 (11%)	1 (1%)	
Missing	84 (47%)	46 (46%)	38 (48%)	
MSI status				0.35
MSI high	1 (1%)	1 (1%)	0	
MSI high not detected	28 (15%)	13 (13%)	15 (19%)	
Undetermined	151 (84%)	86 (86%)	65 (81%)	

AMA: actionable molecular alteration; F1LCDx: FoundationOne Liquid CDx; MA: molecular alteration; MMT: molecularly matched therapy; TT: targeted therapy. * *p*-value is provided for comparison between groups: “AMA” vs. “non-AMA”.

**Table 3:** Efficacy parameters.

Efficacy	AMA + MMT *n* = 37	AMA + Non-MMT *n* = 37	*p*-Value *
PFS2/PFS1			
Median (range)	0.63 (0–11.9)	0.84 (0–7.2)	-
Ratio ≥ 1.3	7/35 (20%)	8/34 (24%)	0.72
Missing	2	3	-
PFS2			0.17
Median, months (95%CI)	2.7 (1.3–3.9)	3.8 (2.1–5.5)	
OS			0.64
Median, months (95%CI)	6.9 (5.0–8.9)	7.2 (4.3–10.1)	
Tumor Response			
Complete response	1 (3%)	0	-
Partial response	6 (16%)	6 (16%)	-
Stable disease	5 (14%)	12 (32%)	-
Progressive disease	17 (46%)	8 (22%)	-
ORR	7 (19%)	6 (16%)	0.87
Disease control	12 (32%)	18 (49%)	0.05
Not available	8 (22%)	11 (30%)	-

AMA: actionable molecular alteration; MMT: molecularly matched therapy; ORR: overall response rate; OS: overall survival; PFS: progression-free survival. * *p*-value is provided for comparison between groups: “AMA + MMT” vs. “AMA + non-MMT”.

## Data Availability

Not available due to privacy issues.

## Supplementary Materials

The following supporting information can be downloaded at: https://www.mdpi.com/article/10.3390/curroncol29030155/s1, Figure S1: Proportions of tumor types. Figure S2: Molecular alterations according to the 4 most common tumor types: (A) Lung cancer (39 most frequent), (B) Breast cancer, (C) Melanoma, and (D) Pancreatic cancer. Figure S3: Incidence of molecular alterations according to tumor type. Figure S4: Kaplan–Meier curves of overall survival (OS) according to PFS2/PFS1 ratio. Table S1: Targeted molecular alterations and molecularly matched therapy used. Table S2: Efficacy parameters in the cohort of patients with level 1-2 AMA (according to OncoKB database [34]) (*n* = 50).
