# Supplementary material for: Clinical Impact of High Throughput Sequencing on Liquid Biopsy in Advanced Solid Cancer
Source: Curr Oncol. 2022 Mar 10;29(3):1902–18. doi: 10.3390/curroncol29030155 (PMC8947301; doi:10.3390/curroncol29030155)
Supplement: Supplementary file 1 [file curroncol-29-00155-s001.zip › Supplementary Materials_reviewed.pdf]

Clinical Impact of High Throughput Sequencing on Liquid Biopsy in Advanced Solid Cancer

SUPPLEMENTARY MATERIALS

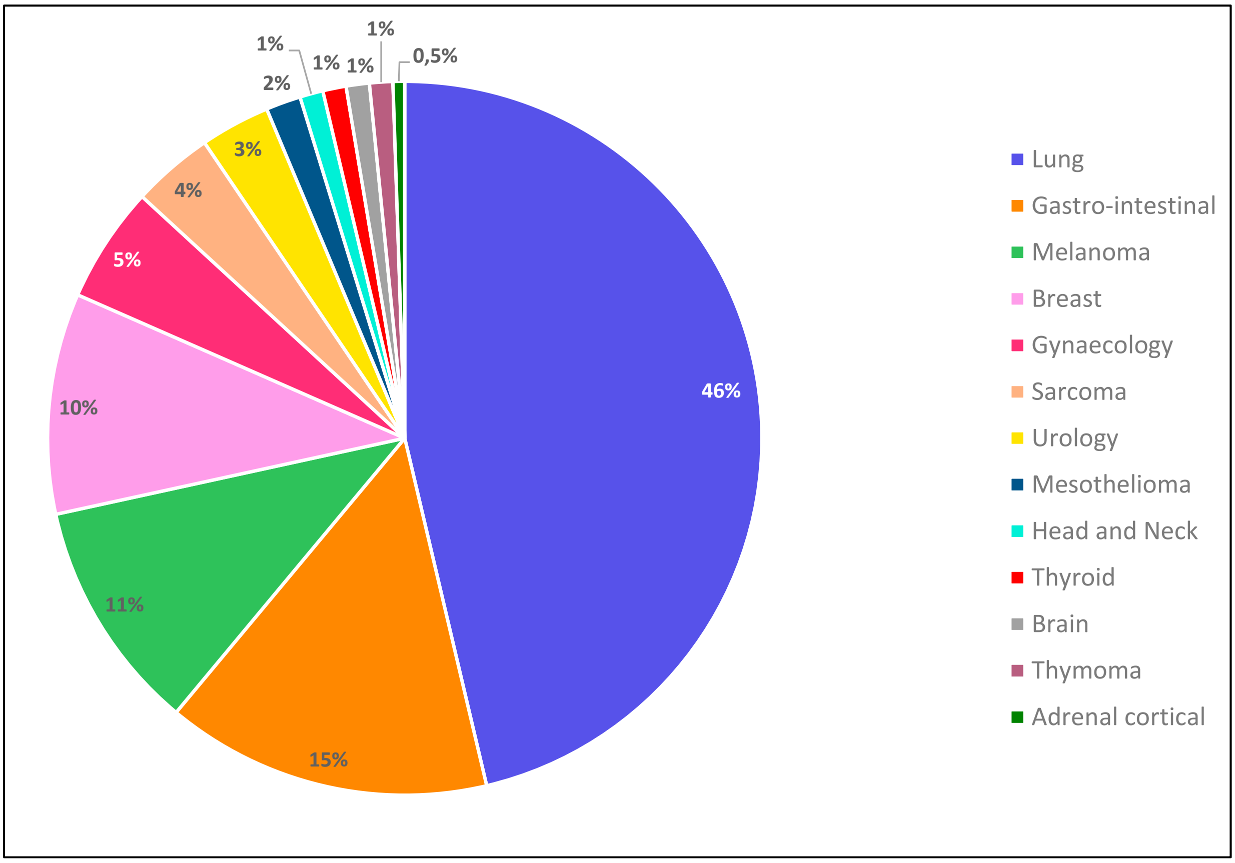

Figure S1: Proportions of tumor types.

Gastro-intestinal tumors include: pancreas (6%), colorectal (4%), stomach (3%), oeosophagus (1%), and cholangiocarcinoma (1%). Gynaecological tumors include: endometrial (3%), ovarian (2%), and cervix (0.5%). Urological tumors include: prostate (2%), kidney (1%), and urinary tractus (0.5%).

Table S1: Targeted molecular alterations and molecularly matched therapy used.

Altered gene	N (%) *(n = 37)*	Specific alteration	VAF (%)	Cancer type	Targeted therapy used
EGFR	5 (14%)	*T790M*	18.9%	Lung	osimertinib
		T790M	0.17%	Lung	osimertinib
		Deletion *exon 19*	20.2%	Lung	osimertinib
		Splice site *2947-2A>C*	6.8%	Lung	afatinib
		Amplification	Detected	Lung	osimertinib
ATM	3 (8%)	*R3008C*	0.15%	Lung	olaparib
		R3008H	-	Colorectal	olaparib + ceralasertib
		Deletion *exons 39-46*	22%	Melanoma	olaparib + dabrafenib + trametinib
BRAF	3 (8%)	*K601N*	5.7%	Lung	dabrafenib + trametinib
		G466R	2.1%	Urinary tractus	dabrafenib + trametinib
		V600E	9.3%	Melanoma	dabrafenib + trametinib
MET	3 (8%)	Amplification	Detected	Lung	crizotinib
		Amplification	Detected	Lung	crizotinib
		Splice site *exon 14* *(3028+3_3028+12delATATTTCAGT)*	0.38%	Lung	capmatinib
PTEN	3 (8%)	*G127**	30.7%	Melanoma	everolimus
		PTEN loss	Detected	Breast	everolimus + exemestane
		*K183fs*16*	11.2%	Breast	everolimus
PIK3CA	3 (8%)	*E545K*	1.1%	Lung	everolimus
		E542K	15.7%	Breast	olaparib + fulvestrant + gosereline
		E545K	1.5%	Breast	everoliumus + exemestane
CHEK2	2 (5%)	*L96**	-	Lung	olaparib + ceralasertib
		*R523fs*43*	0.24%	Stomach	rucaparib + atezolizumab
TMB	2 (5%)	19 mut/Mb	-	Pancreas	pembrolizumab
		95 mut/Mb	-	Stomach	nivolumab
PALB2	1 (2%)	*S779*	49.7%	Lung	olaparib
BRCA2	1 (2%)	*R1738fs*2*	50.9%	Prostate	olaparib
GNAQ	1 (2%)	*Q209L*	4.7%	Melanoma	trametinib

GNA11	1 (2%)	*Q209L*	0.52%	Melanoma	trametinib
HRAS	1 (2%)	*Q61R*	22.3%	Lung	tipifarnib
CDKN2A/B	1 (2%)	*p16INK4a W15**	6.8%	Melanoma	abemaciclib
MTOR	1 (2%)	*T1977R*	11.9%	Lung	everolimus
RET	1 (2%)	*V804M*	48.8%	Lung	selpercatinib
ERBB2	1 (2%)	Amplification	Detected	Breast	trastuzumab-emtansine
ESR1	1 (2%)	*Y537N*	-	Breast	fulvestrant
TSC1	1 (2%)	*Q280**<br>*L627fs*22*	8.8%<br>1.2%	Breast	everolimus + exemestane
ROS1-FGD	1 (2%)	Fusion (non-canonical)	4.1%	Lung	lorlatinib
EML4-ALK	1 (2%)	Fusion (variant 1)	1.1%	Lung	Alectinib

TMB: Tumor mutational burden; VAF: Variant allelic frequency

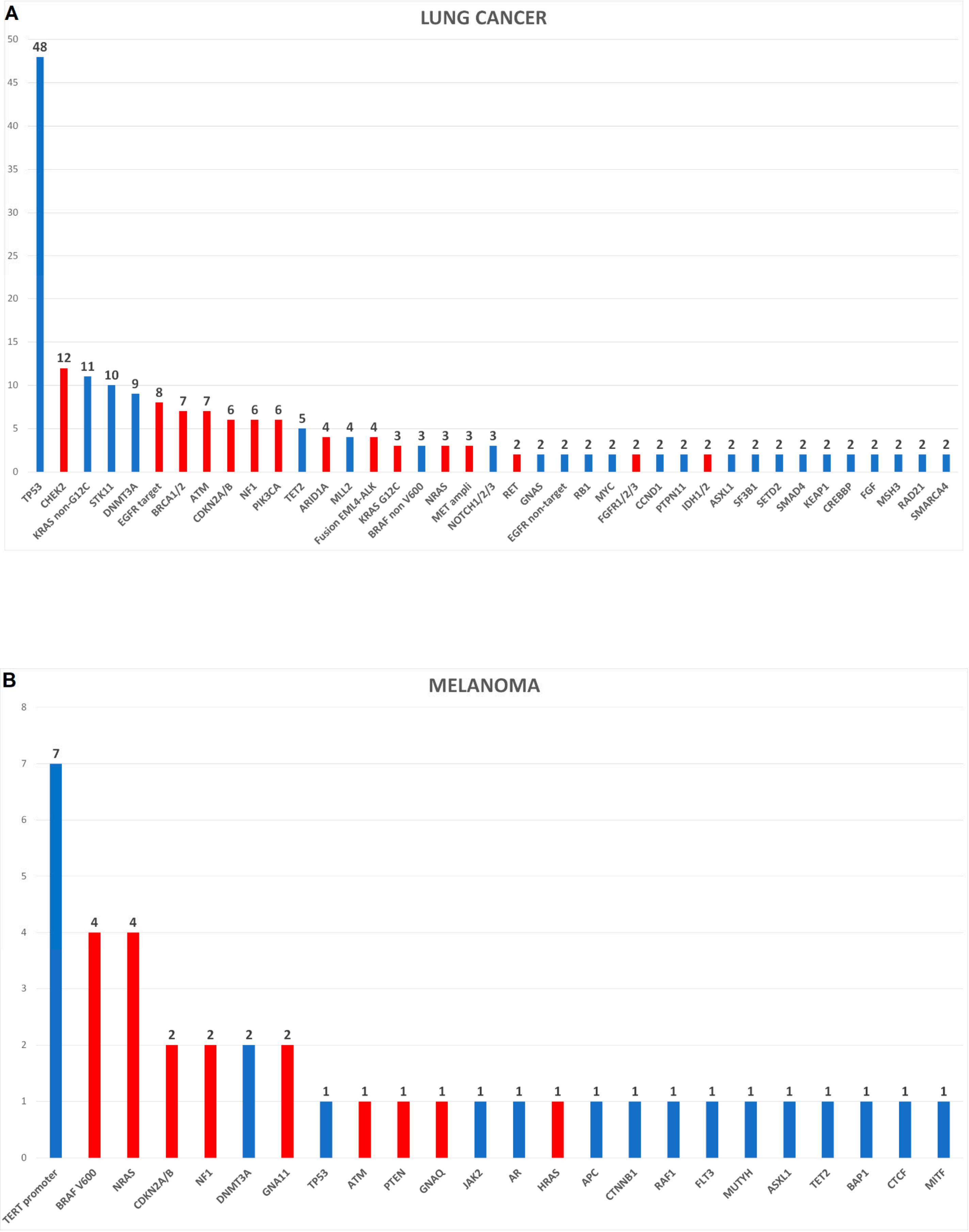
A
LUNG CANCER
TP53 48
CHEK2 12
KRAS non-G12C 11
STK11 10
DNMT3A 9
EGFR target 8
BRCA1/2 7
ATM 7
CDKN2A/B 6
NF1 6
PIK3CA 6
TET2 5
ARID1A 4
MLL2 4
Fusion EML4-ALK 4
KRAS G12C 3
BRAF non V600 3
NRAS 3
MET ampli 3
NOTCH1/2/3 3
RET 2
GNAS 2
EGFR non-target 2
RB1 2
MYC 2
FGFR1/2/3 2
CCND1 2
PTPN11 2
IDH1/2 2
ASXL1 2
SF3B1 2
SETD2 2
SMAD4 2
KEAP1 2
CREBBP 2
FGF 2
MSH3 2
RAD21 2
SMARCA4 2
B
MELANOMA
TERT promoter 7
BRAF V600 4
NRAS 4
CDKN2A/B 2
NF1 2
DNMT3A 2
GNA11 2
TP53 1
ATM 1
PTEN 1
GNAQ 1
JAK2 1
AR 1
HRAS 1
APC 1
CTNNB1 1
RAF1 1
FLT3 1
MUTYH 1
ASXL1 1
TET2 1
BAP1 1
CTCF 1
MITF 1

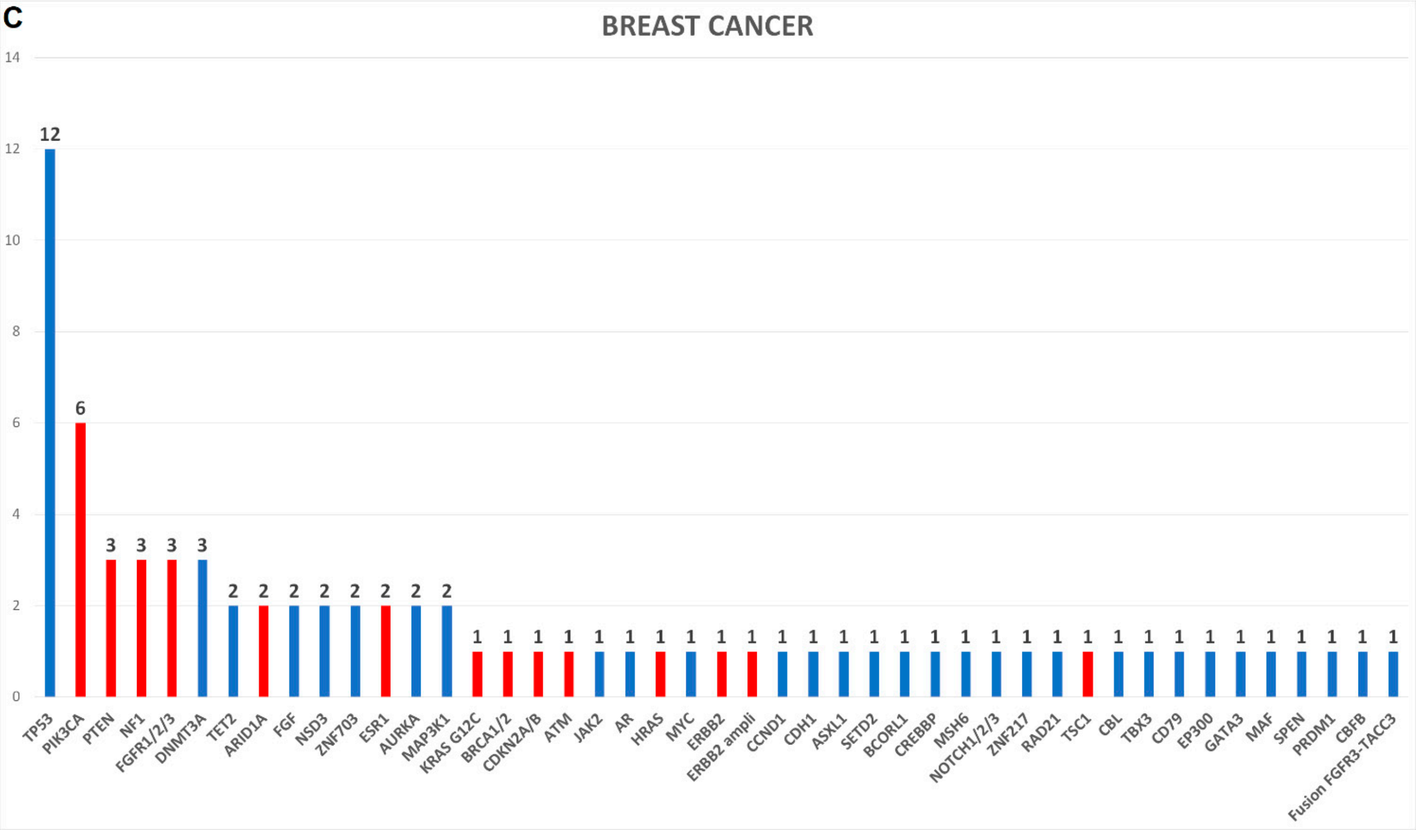

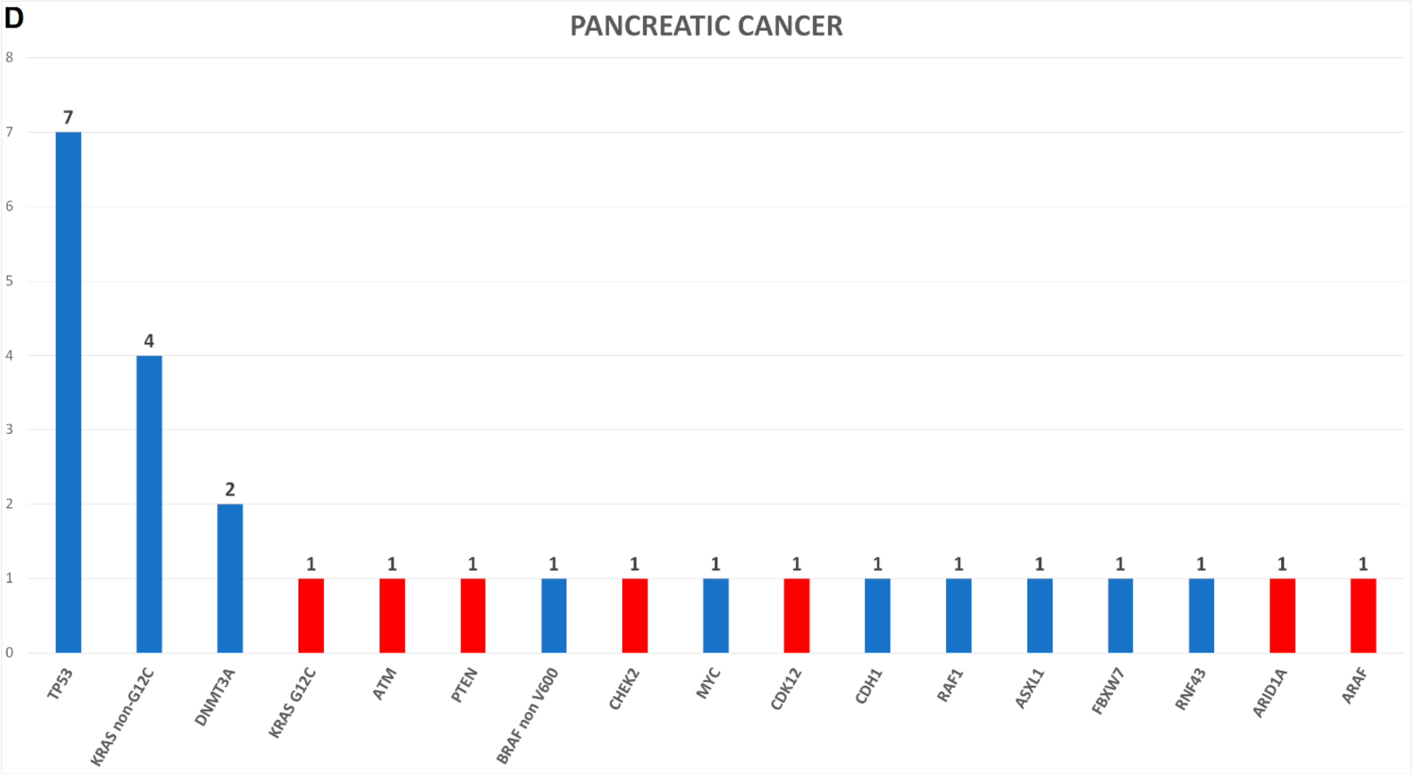

Figure S2: Molecular alterations according to the 4 most common tumor types: **(A)** Lung cancer (39 most frequent), (**B**) Breast cancer, **(C)** Melanoma, and **(D)** Pancreatic cancer.

Actionable molecular alterations are colored in red. Non-actionable molecular alterations are colored in blue.

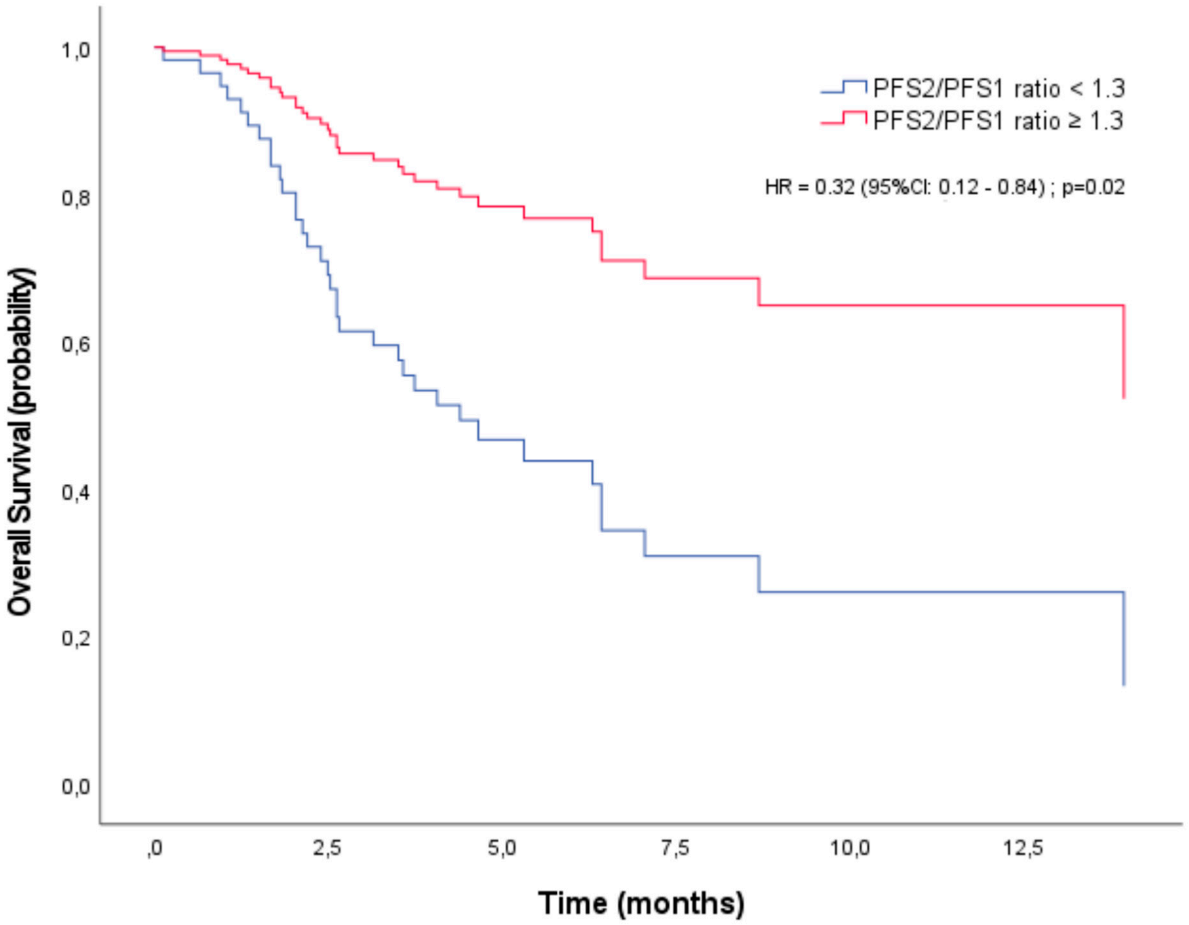

Figure S4: Kaplan-Meier curves of overall survival (OS) according to PFS2/PFS1 ratio.

Table S2: Efficacy parameters in the cohort of patients with level 1-2 AMA (according to OncoKB database [34]) (*n=50*).

**Efficacy**	**Level 1-2 AMA + MMT** *n=23*	**Level 1-2 AMA + non-MMT** *n=27*	***p*-Value***
PFS2/PFS1			
Median (range)	0.63 (0 – 11.9)	0.81 (0 – 7.2)	-
Ratio ≥ 1.3 (%)	4/21 (19%)	7/26 (27%)	0.73
Missing	2	1	-
PFS2			0.87
Median (95%CI)	2.7 (0.7 – 4.7)	2.8 (2.3 – 3.2)	
OS			0.60
Median (95%CI)	4.7 (1.4 – 7.9)	7.2 (0.0 – 14.5)	
Tumor response			
Complete response	1 (4%)	0	-
Partial response	4 (17%)	3 (11%)	-
Stable disease	4 (17%)	7 (26%)	-
Progressive disease	9 (39%)	11 (41%)	-
ORR	5 (22%)	3 (11%)	0.43
Disease control	9 (39%)	10 (37%)	1.00
Not available	5 (22%)	6 (22%)	-

AMA: actionable molecular alteration; MMT: molecularly matched therapy; ORR: overall response rate; OS: overall survival; PFS: progression-free survival

* *p*-Value is provided for comparison between groups: “Level 1-2 AMA + MMT” *vs* “Level 1-2 AMA + non-MMT”
